# Vibration Sensor Based on Hollow Biconical Fiber

**DOI:** 10.3390/s21041023

**Published:** 2021-02-03

**Authors:** Yingfang Zhang, Ben Xu, Dongning Wang, Yun Guo, Min Chen, Weicheng Chen, Penglai Guo, Xiaoling Peng, Jianqing Li

**Affiliations:** 1 Faculty of Information Technology, Macau University of Science and Technology, Avenida Wai Long, Taipa, Macau, China; 1909853eii20002@student.must.edu.mo (Y.Z.); 2009853yii30003@student.must.edu.mo (P.G.); 2009853Cii30001@student.must.edu.mo (X.P.); jqli@must.edu.mo (J.L.); 2 The College of Optical and Electronic Technology, China Jiliang University, No. 258, Xueyuan Street, Xiasha Higher Education Zone, Hangzhou 310018, China; dnwang@cjlu.edu.cn (D.W.); S1904080304@cjlu.edu.cn (Y.G.); P1804085206@cjlu.edu.cn (M.C.); 3 Guangdong-Hong Kong-Macao Joint Laboratory for Intelligent Micro-Nano Optoelectronic Technology, Foshan University, Foshan 528225, China; chenwch@fosu.edu.cn

**Keywords:** optical fiber sensor, hollow biconical fiber, interferometer, vibration, temperature

## Abstract

A hollow biconical fiber is proposed and experimentally demonstrated for vibration sensing. It is fabricated by creating an air micro-cavity in single-mode fiber, followed by tapering it. Experimental results show that the device is highly sensitive to bending with a sensitivity of 21.30 dB/m^−1^. When it is exposed to vibration, its transmission loss is modulated periodically, then based on the measured transmission, the vibration frequency can be demodulated accurately. The acoustic vibration testing results show that the proposed device can detect and demodulate the exciting acoustic frequency accurately and distinguish its sound intensity, and the maximum signal to noise ratio (SNR) achieves up to 59 dB. Moreover, cantilever beam testing proves its performance reliable. Additionally, the sensing head has the advantages of a lightweight, compact size (with a total length of less than 250 μm), and insensitivity of temperature. All these features indicate the proposed sensor has a promising potential in the engineering field.

## 1. Introduction

Vibration is a common phenomenon in nature, but it often causes industrial engineering damage [1]. Compared with mechanical sensors and piezoelectric sensors, optical fiber vibration sensors are more suitable for vibration measurement owing to their advantages, such as small size, anti-electromagnetic interference, lightweight, high sensitivity, optical fiber sensing is widely used in aeronautical [2,3], structural health [2,4,5,6], environmental monitoring [7], and even harsh environments [8,9]. Therefore, it is necessary to detect the vibration accurately and effectively by a sensor.

Optical fiber sensors have been applied to the field of vibration sensing. Many structures have been used in the field of vibration sensing, such as an optical fiber ring structure [10,11], Fiber Bragg grating [12,13], in-line interferometer [14,15,16], and distributed vibration sensor [17]. Among them, various interferometers with a sandwich structure formed by combining different types of fibers are extensively adopted owing to the features of easy fabrication, high sensitivity, and a wide frequency response range. A vibration sensor formed by splicing a segment of multicore fiber (MCF) to a single-mode fiber (SMF) is a typical example, and it has a large frequency response range of several hertz to several kilohertz [18]. However, it has a long length of several tens of millimeters. Some small-sized structures have been proposed. For example, a Fabry-Perot interferometer fabricated by hollow-core fiber (HCF), SMF, and coreless silica fiber (CF) have been proposed. Its sensitivity is up to 20.678 mV/g at a frequency of 500 Hz, and the frequency range of vibration sensing is as wide as a range from 100 Hz to 3000 Hz. Unfortunately, although the size of this structure is small, it uses a lot of materials, high cost, and a complex manufacturing process [19]. A butterfly-shaped sensor with a sandwich structure is composed of a smaller size, and lower cost is proposed. The SMF-HCF-SMF structure is used to measure low-frequency vibration. Its sensing element is a tapered hollow-core fiber with a length of 595 μm, which measured low-frequency vibration of 10–200 Hz with a poor error rate of 0.27% [20]. A Mach-Zehnder interferometer composed of bending-insensitive fiber (BIF) solved the problem of complex structure. The BIF-MZI structure measured continuous vibration frequencies from 1 Hz to 500 kHz, but it still has a larger size with the length of the taper is 900 μm [21].

In this paper, we proposed another new vibration sensor based on hollow biconical fiber (HBF). To fabricate it, only commercial communication SMF is required, and the total length of the sensing head is less than 1 mm. Experimental results showed that the sensing head has a high bending sensitivity of 21.3 dB/m^−1^, and owing to this feature, the sensing head can be used for sensing surrounding vibration frequency and amplitude with high SNR. A cantilever beam testing further proves the performance of it reliable.

## 2. Principle and Fabrication

**Figure 1:**
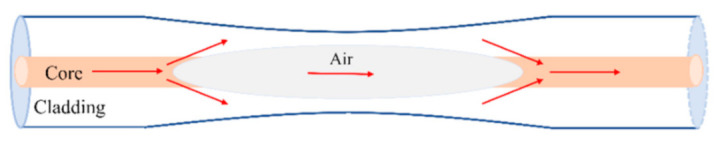
Schematic diagram of the proposed sensing structure.

The structure diagram of the proposed device is shown in Figure 1. An air micro-cavity was embedded in a biconical fiber to form a hollow taper. Launched light from a broadband light source was coupled into the core of lead-in SMF and then split into two beams due to the spherical interface of silica core/air, one called as cavity mode propagates in the cavity and the other called as cladding mode in the cavity wall, then they combined in the lead-out SMF core. Because of the optical path difference (OPD) caused by different mediums in two paths, a phase difference is caused between these two beams; thus interference is generated, and the configuration of the proposed device can be considered as a Mach-Zehnder interferometer (MZI).

According to Fermat’s Lemma [22], the OPD can be expressed as $OPD=L\Delta n_{eff}$, where L indicates the interference length, $\Delta n_{eff}=n_{eff}^{cladding}-n_{eff}^{cavity}$ is the difference between the effective refractive index of cladding and air. The output light intensity can be expressed as:

(1) $$I=I_{Cavity}+I_{Cladding}+2\sqrt{I_{Cavity}I_{Cladding}}\cos\left(\frac{2\pi L\Delta n_{eff}}{\lambda }\right),\;$$

where $I_{Cavity}$ and $I_{Cladding}$ indicate the intensity of light propagating in the air cavity and fiber cladding, respectively, and λ is the wavelength of light in free space. When the phase difference $\Delta \varphi \left(=\cos\left(\frac{2\pi L\Delta n_{eff}}{\lambda }\right)\right)$ satisfies the condition of $\Delta \varphi =\left(2m+1\right)\pi$, the output light intensity achieves to a minimum value corresponding to a dip in the transmitted spectrum of the device.

If the biconical fiber is modulated by some external factors, such as temperature, vibration, or bending, its transmission spectrum may shift accordingly. Calibrating the functional relationship between them, the proposed device can be used for sensing this physical parameter.

**Figure 2:**
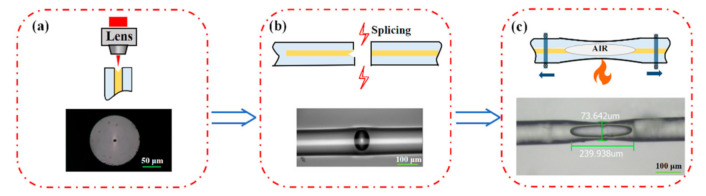
Fabrication process of the proposed sensing structure based on a hollow biconical fiber (HBF). (**a**) Micro-pit drilling by fs-laser, (**b**) Fusion splicing to form an inner air micro-cavity, and (**c**) Tapering by flame to form an HBF.

**Figure 3:**
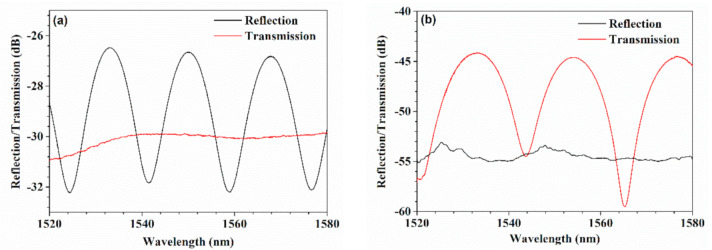
Spectra of the fiber with an inner air-cavity (**a**) before and (**b**) after tapering.

To fabricate the biconical fiber as described above, several steps were involved. First, a small pit was drilled in the end-face of a SMF cleaved flat using a focused femtosecond laser, as shown in Figure 2a, and the inset shows the micrograph of the drilled SMF. The pit had a diameter of about 6 μm with a depth of 10 μm. Then, the drilled SMF was spliced to another SMF with a flat end-face using a fiber splicing machine (FSM-80S, Fujikura, Tokyo, Japan) in the standard SMF-SMF splice mode. Then, an air micro-cavity was formed in the fiber with a cavity length of ~70 μm, as shown in Figure 2b. Figure 3a presents the reflection and transmission spectra of the fiber with an inner air-cavity. It can be seen that there were obvious fringes in the reflection spectrum, while the interference fringes could hardly be seen in the transmission spectrum, which can be explained by the Fabry–Perot interference (FPI) due to the air-cavity acts as an F-P cavity. In further, according to the free spectrum range (FSR) of 17.34 nm in the reflection spectrum, the cavity length was calculated to be 69.3 μm, which agreed well with the measured value. Finally, to taper the fiber with an inner air-cavity by flame heating, as shown in Figure 2c. Heating the air cavity with oxyhydrogen flame and stretching the fiber longitudinally to both sides, an HBF was then obtained with features of 225 μm cavity length and 73.6 μm waist diameter, and the thinnest cavity wall at the taper waist had a thickness of ~15.4 μm. Figure 3b presents the reflection and transmission spectra of the air-cavity after tapering, and it is worth noting that the interference fringes having existed in the reflection spectrum disappeared, while there were obvious fringes in the transmission spectrum, which can be explained by the fact that FPI was destroyed and MZI was constructed due to the tapering process. According to the FSR of 22.4 nm in the transmission spectrum, the interference length was calculated to be ~229.1 μm, which was close to the measured value.

## 3. Experiments and Discussions

### 3.1. Bending Response

**Figure 4:**
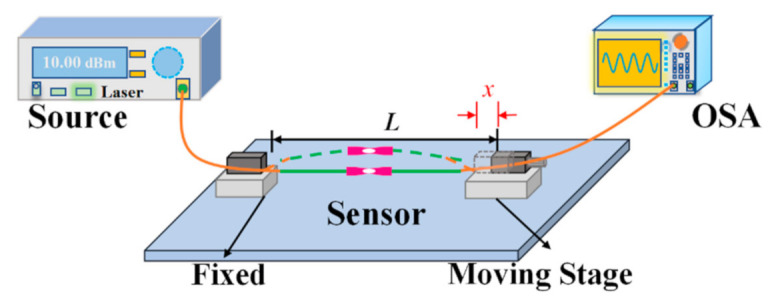
Experimental system for bending sensing.

First, the bending response of the device was tested, and the experimental setup is shown in Figure 4. The HBF was pasted on the surface of a steel ruler, and the ruler was mounted between a fixed stage and a high-resolution translation stage with an initial distance of $L_{0}$ (=200 mm), and one end of the HBF was connected to an amplified spontaneous emission (ASE, ALS-CL-17-B-FA, Amonics, Hong Kong, China) covering a wavelength range of 1520–1580 nm, the other to an optical spectrum analyzer (OSA, AQ6370D, Yokogawa, Japan) with the highest resolution bandwidth of 0.02 nm. When the translation stage moved to the left, the ruler bends with the HBF attached to it obtained a certain curvature. And the curvature can be evaluated by the formula $C=1/R=\sqrt{24x/L_{0}^{3}}$ [23], where R is the bending radius of the fiber and x denotes the displacement of the moving stage.

**Figure 5:**
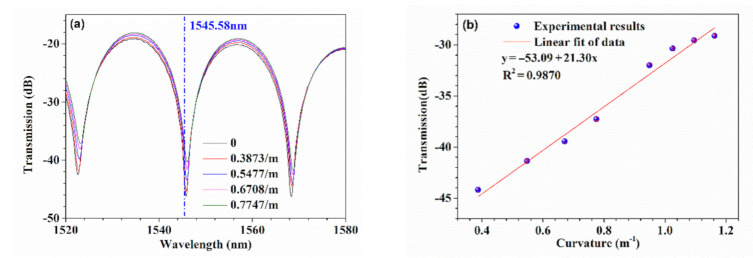
Bending response of the proposed sensing structure. (**a**) Transmission spectra of the device with typical curvatures. (**b**) The relationship between the transmission and the curvature of the device for a single wavelength light.

Figure 5a shows the transmission spectra of the HBF with typical curvatures. It can be seen that the transmission spectrum exhibited a red shift with the increasing curvature. For the light with a wavelength of 1545.58 nm near the resonance dip, its transmission was modulated by the curvature of the fiber, showing a monotonous change with the increase in curvature. Figure 5b gives the detailed transmissions with error bars under different curvatures. The least-square linear fitting method was adopted, and it was found that there was a good linear relationship between them with an *R*^2^ of 0.9870. The slope of the fit function was up to 21.30 dB/m^−1,^ implying the device was highly sensitive to bending.

### 3.2. Acoustic Vibration Testing

**Figure 6:**
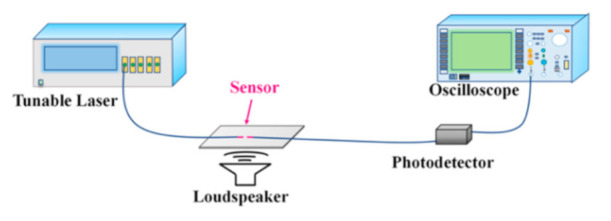
Schematic setup for the acoustic vibration sensing.

Based on the high bending sensitivity of the HBF, an acoustic vibration testing setup was constructed, as shown in Figure 6, and the vibration response of the device was investigated. The HBF was pasted on a square steel plate with a side length of 100 mm and 0.6 mm thickness, and one end of the HBF was connected to a tunable laser covering a wavelength range of 1520 to 1570 nm with a wavelength resolution of 10 pm, the other to an InGaAs biased photodetector with a wide dynamic range and high sensitivity connecting to an oscilloscope with data storage function. A loudspeaker was placed below the steel plate, and the acoustic wave drove the plate with the attached HBF to vibrate, and thus the HBF periodically bent. In our experiments, the tunable laser was tuned to 1544.57 nm, which corresponds to a steep resonance region with a large slope.

**Figure 7:**
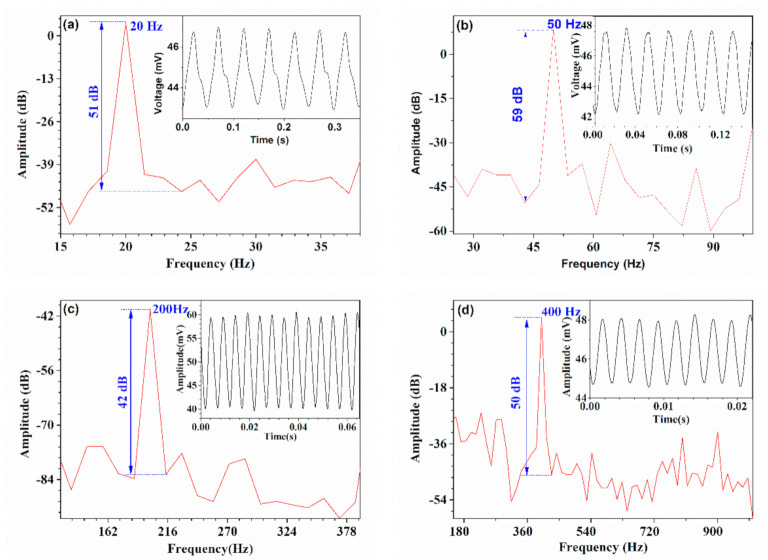
Demodulation of the vibration frequency based on the time-domain spectrum of the ouput voltage signal of the HBF driven by acoustic wave with different frequencies of (**a**) 20 Hz, (**b**) 50 Hz, (**c**) 200 Hz, and (**d**) 400 Hz, respectively.

Figure 7 presents the testing results for acoustic vibration with typical frequencies. The loudspeaker was successively driven by sinusoidal voltages with 20 Hz, 50 Hz, 200 Hz, and 400 Hz. The black solid lines in insets give the output voltage signals of the photodetector in the time-domain when the HBF was exposed to vibration. Obviously, the output voltage signals vary periodically. Then, fast Fourier transform (FFT) was adopted to the time-domain signals to demodulate the vibration frequency, and the red lines in Figure 7 denote the FFT results. It was found that the demodulated frequencies exactly agreed with the driving acoustic frequencies with a maximum signal to noise ratio (SNR) of 59 dB, which implies the HBF can be used for demodulating acoustic frequencies accurately.

**Figure 8:**
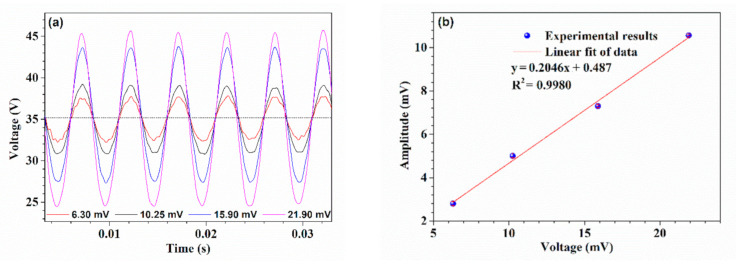
Response of the device based on HBF to vibrations amplitude. (**a**) Time-domain spectra of the output voltage, (**b**) Half peak–peak amplitudes of the output voltage at different driving voltages.

Further, the response of the device to the vibration amplitude was tested. The sinusoidal voltages driving the loudspeaker was kept at a constant frequency of 200 Hz while its amplitude was adjusted, and the output signal of the photodetector was recorded in real-time. Figure 8a presents the measured time-domain spectra of the output voltage, and for clarity and readability, the signals were shifted slightly on the time axis, but it did not affect the analysis of the data. Figure 8b gives the detailed half peak–peak amplitudes of the output voltage with error bars at different driving voltages. Similarly, the least square linear fitting method was adopted. Obviously, there was a good linear relationship between them with *R*^2^ of 0.9980, and the slope indicates the amplitude sensitivity, which implies the device based on HBF is capable of distinguishing the vibration amplitude. It should be noted that the amplitude sensitivity relates to the dimensions of the HBF, such as the taper length, the waist diameter, the profile of the ellipsoidal air micro-cavity, which determines the number and order of cladding modes propagated in the cavity wall. And it is believable that the fiber taper with a thinner waist is easier to bend, i.e., it is more sensitive to bending, but inevitably, it is more fragile. Then, there is a tradeoff between sensitivity and mechanical robustness. To optimize the dimension of the HBF is our future work.

### 3.3. Cantilever Beam Testing

**Figure 9:**
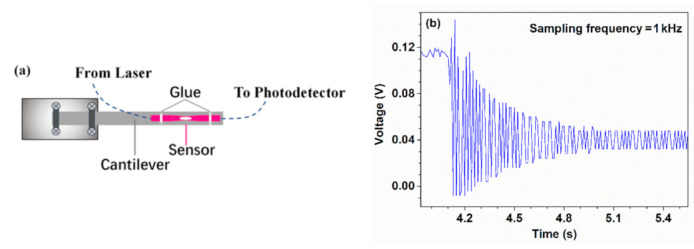
(**a**) Schematic of the experimental setup for damped vibration testing. (**b**) A typical time-domain spectrum of the output voltage when the HBF was exposed to damped vibration.

Then, the HBF was used for cantilever beam testing. Figure 9a shows the schematic of the experimental setup. The HBF was pasted on a stainless-steel ruler, and its two ends were connected to a tunable laser and an oscilloscope through a photodetector, respectively. The ruler had a rectangular cross-section with a length of 20.0 mm and thickness of 0.6 mm. One end of the ruler was mounted on the edge of an optical table, and the other end was suspended to form a cantilever. When the free end of the cantilever was deflected by a specific displacement from its initial stabilized position and instantly released, the cantilever beam would experience a damped vibration about its equilibrium position soon afterward. Further, the damped natural frequency of the cantilever can be evaluated following the formula: $f=\frac{c}{2\pi }\sqrt{\frac{EI}{\rho AL^{4}}}$ [24], where *c* is the coefficient of the first vibration mode, *E* is Young’s modulus of stainless steel, *L*, *ρ*, and *A* are the length, density, and the cross-sectional area of the cantilever, respectively, *I* denotes the moment of inertia, and it can be expressed as $ab^{3}/12$ since the cross-section of the cantilever beam was rectangular, where a and b correspond to the width and thickness of the used ruler, respectively. Figure 9b shows a typical time-domain spectrum of the output voltage while the HBF was exposed to damped vibration caused by a cantilever beam with a certain length. It can be seen that the oscillation amplitude gradually decreased as time increases.

**Figure 10:**
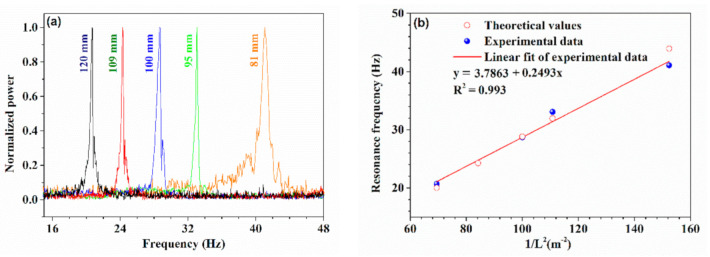
(**a**) Normalized power spectra with different cantilever lengths. (**b**) Fundamental frequencies as a function of cantilever lengths

Figure 10a shows the normalized FFT results of the output voltage in the time domain for damped vibration under different cantilever beam lengths, in which the peaks correspond to the fundamental frequency for each case. It can be seen that with the increase in cantilever length, the fundamental frequency decreased. The measured four fundamental frequency were 41.26 Hz, 33.07 Hz, 28.71 Hz, 24.29 Hz, and 20.71 Hz for cantilever length of 81 mm, 95 mm, 100 mm, 109 mm, and 120 mm, and they were very close to the theoretical values of 43.94 Hz, 31.95 Hz, 28.84 Hz, 24.28 Hz, and 20.02 Hz, respectively. Furthermore, the linear fitting results show that there was a very good linear relationship between the fundamental frequency and $1/L^{2}$ with $R^{2}$ of 0.9993, as shown in Figure 10b, which agreed well with the theoretical analysis above. The experimental results of the cantilever beam testing prove the performance reliability of our proposed HBF.

### 3.4. Temperature Response

**Figure 11:**
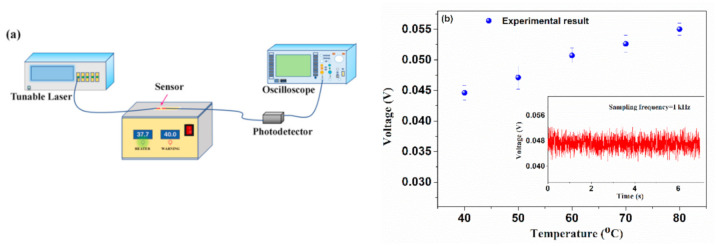
(**a**) Schematic setup for temperature response measurement of the proposed device and (**b**) the temperature response of the device. Inset: transmitted light power in time-domain at a constant temperature of 50 °C.

Finally, the effect of temperature on the HBF was tested. The schematic setup is shown in Figure 11a. The sensor was placed on a platform whose temperature can be controlled with a resolution of 0.1 °C within the range of 40 °C to 80 °C, and the output voltage indicating the variation of transmitted light through the sensor was monitored by an oscilloscope. Figure 11b shows the average output voltage at different temperatures. It was found that the intensity of transmitted light slightly increased with a small fluctuation as the temperature of HBF increased. The inset in Figure 11b shows the monitoring results over a short period, keeping a constant temperature of 50 °C. Obviously, the output voltage was almost kept a constant with a small and irregular fluctuation, which may come from the uncertainty of temperature control or the instability of the laser source. Generally, if the surrounding temperature is a slow-moving and aperiodicity variable, it has little effect on the demodulation of vibration frequency.

## 4. Conclusions

An HBF was proposed and experimentally demonstrated for vibration sensing. It was fabricated by tapering an SMF with an inner micro-cavity, which was obtained by combining femtosecond laser micro-machining and fiber fusion splicing technology. Due to the high bending sensitivity of the HBF, it can be used for vibration sensing. Experimental results showed the sensor is capable of correctly demodulating surrounding vibration frequency with a high SNR and distinguishing the vibration amplitude. Moreover, the ambient temperature had little effect on its performance. To fabricate the sensor, no special fibers were required, but only commercial SMF, and the sensing head had a total length of less than 250 μm. All these features imply the proposed HBF has a promising potential for vibration sensing in the engineering fields.
